# Women’s empowerment and child growth faltering in Ethiopia: evidence from the Demographic and Health Survey

**DOI:** 10.1186/s12905-021-01183-x

**Published:** 2021-01-30

**Authors:** Alemayehu Gonie Mekonnen, Daniel Bogale Odo, Dabere Nigatu, Adem Sav, Kiya Kedir Abagero

**Affiliations:** 1 College of Health Science, Debre Berhan University, Debre Berhan, Ethiopia; 2 College of Health Sciences, Arsi University, Asela, Ethiopia; 3 School of Public Health, College of Medicine and Health Sciences, Bahir Dar University, Bahir Dar, Ethiopia; 4 School of Public Health and Social Work, Faculty of Health, Queensland University of Technology, Brisbane, QLD Australia; 5 Non Communicable Disease Directorate, Armauer Hansen Research Institute, Addis Ababa, Ethiopia

**Keywords:** Women’s empowerment, Child growth faltering, EDHS

## Abstract

**Background:**

Despite numerous national and international efforts to alleviate child growth faltering, it remains a global health challenge. There is a growing body of literature that recognizes the importance of women’s empowerment in a wide range of public health topics, such as the utilization of maternal healthcare services, agricultural productivity, and child nutrition. However, in Ethiopia, the relationship between women’s empowerment and child nutritional status is not studied at the national level. This study aimed to determine the association between women’s empowerment and growth faltering in under-5 children in Ethiopia.

**Methods:**

The data source for this analysis is the 2016 Ethiopian Demographic and Health Survey (EDHS): a nationally representative household survey on healthcare. The EDHS employed a two-stage stratified cluster sampling technique. We computed standard women’s empowerment indices, following the Survey-based Women’s emPowERment index approach. A multilevel logistic regression model that accounted for cluster-level random effects was used to estimate the association between women’s empowerment and child growth faltering (stunting, wasting and underweight).

**Results:**

Attitude to violence, social independence, and decision-making were the three domains of women’s empowerment that were associated with child growth faltering. One standard deviation increase in each domain of empowerment was associated with a reduction in the odds of stunting: attitude towards violence (AOR = 0.92; 95% CI 0.88–0.96; *p* < 0.001), social independence (AOR = 0.95; 95% CI 0.89–0.99; *p* = 0.049), and decision-making (AOR = 0.93; 95% CI 0.87–0.99; *p* = 0.023). Similarly, each standard deviation increase in attitude towards violence (AOR = 0.93; 95% CI 0.89–0.98; *p* = 0.008), social independence (AOR = 0.91; 95% CI 0.86–0.97; *p* = 0.002), and decision-making (AOR = 0.92; 95% CI 0.86–0.99; *p* = 0.020) were associated with a decrease in the odds of having underweight child.

**Conclusions:**

Ensuring women’s empowerment both in the household and in the community could have the potential to decrease stunting and underweight in a rapidly developing country like Ethiopia. Policymakers and health professionals need to consider women’s empowerment in this unique context to improve nutritional outcomes for children and alleviate growth faltering.

## Introduction

In most societies, women are predominantly responsible for the selection, procurement, preparation, and provision of food for children in the household [1]. They play an important role in child feeding and make significant investments for their children [2]. As a result, the nutritional status of children is largely influenced by women’s characteristics, including their level of empowerment at the household level and in the community.

Women’s empowerment is a process and multidimensional by nature, and the concept operates at various levels [3]. Although there is no single commonly accepted definition of women’s empowerment, most accepted is the ability of a woman to claim enabling resources, exercise voice and agency, and act on desires to transform her own life, in contexts where these abilities have been denied [4, 5]. Different studies considered various sets of indicators to measure different dimensions of women’s empowerment [6–8]. For instance, the Demographic and Health Survey (DHS) based studies which collect and disseminates accurate and nationally representative data on healthcare in developing countries have used two indicators to measure women’s empowerment; decision-making at the household and women’s attitude towards violence [9, 10]. However, the recently published Survey-based Women’s emPowERment (SWPER) index that based on DHS data of 34 African countries included some items that could better approximate the social independence aspect of women’s empowerment [11]. Indeed, there are multifaceted discriminatory social and cultural norms, particularly in developing countries like Ethiopia, that can hinder women’s ability in several ways [12].

There is a growing body of literature that recognizes the importance of women’s empowerment in a wide range of public health topics such as the utilization of maternal healthcare services, agricultural productivity, and child nutrition [13–16]. Based on this evidence, women’s empowerment is more important than household poverty in determining the nutritional status of children, and it influences child nutritional status through a range of mechanisms [10, 17]. If women have freedom of plan of action and can decide on household income, they may have a high influence on household food choice to improve the diet and nutritional status of children [18, 19]. Hence, the lack of women’s empowerment may be an underlying factor that contributes to child malnutrition [7, 20]. Indeed, studies have examined the association between women’s empowerment (despite dissimilarities in the operationalization) and child undernutrition. However, these studies have reported inconsistent results. For example, research from Bangladesh [21] and Benin [22] suggested that women’s empowerment is associated with improved child feeding practices that affect child nutrition. On the other hand, a study from Mozambique [23] and Ghana [24] found no association between domains of women's empowerment and child growth faltering. Despite the mixed results, we could not find a published document on the relationship of different dimensions of women’s empowerment and child nutrition within the Ethiopian context.

In Ethiopia, women’s empowerment status is considered to be predominantly low [25] and food preparation is considered primarily women’s duty [26]. Childhood undernourishment in the country remains highly prevalent. According to the Ethiopian Demographic and Health Survey (EDHS) 2016 report, 38% of children are stunted, 10% are wasted and 24% are underweight [27]. Available evidence revealed that child mortality is about three times higher among women who had low decision-making ability than high decision-makers [28]. However, we do not know whether women’s attitude towards domestic violence, social independence, and decision-making capabilities in the household, which have found to be important to child nutritional outcomes in various studies, actually have an impact on child nutrition in Ethiopia.

The current study aimed to determine the association between women’s empowerment in terms of their attitude towards domestic violence, social independence, and decision-making in the household and growth faltering in Ethiopian children. To the best of our knowledge, there is no nationwide analysis that examines the relationship between such domains of women’s empowerment and growth faltering among Ethiopian children. Indeed, efforts to reduce stunting, wasting and underweight health issues in Ethiopian children will not be fruitful without having adequate knowledge of the relationship between women’s status both in the household and in the community with respect to childhood nutritional status. By understanding such association, our findings may help policymakers design sensitive programs that consider women’s empowerment to alleviate childhood malnutrition in this context.

## Methods

### Data sources and study population

The data source for this analysis is the 2016 EDHS. The DHS is a principal source of data on a wide range of public health topics, including child nutrition and women’s empowerment. This survey followed a two-stage stratified cluster sampling, in which clusters were selected from a list of enumeration areas, primary sampling units, formed for 2007 population census, and then households were randomly selected in each of the selected clusters (secondary sampling unit). Ethiopia is divided into nine geographical regions and two administrative cities. The 2016 EDHS was designed to collect representative samples from each administrative division and urban and rural areas separately. The survey target groups were children aged 0–5 years, women aged 15–49 years, and men aged 15–59 [27]. In the current study, the unit of analysis was children aged less than 5 years.

Five survey modules (questionnaires) were used for the 2016 EDHS. Of those, the woman’s questionnaire was used to collect information from women of reproductive age (15–49 years) about their reproductive history and other child and household-related topics. In addition to the demographic issues, anthropometric information (height and weight) of under-five children were recorded. Details on sampling and data collection procedure of EDHS are described elsewhere [27]. The EDHS is approved by ORC Macro Institutional and by the Ethiopian ethical review board. Authors got permission from ICF-DHS program to use the DHS data, accessed online (http://dhsprogram.com).

### Outcome variable

Child growth faltering indicators: stunting, wasting and underweight were the outcome variables. Each child growth faltering indicator was defined using the anthropometric Z-scores assigned based on the World Health Organization (WHO) Child Growth Standards [29]. Stunting was defined as a height-for-age Z score less than minus two standard deviations (SD) below the median WHO Child Growth reference, wasting as a weight-for-height Z-score less than negative 2 SD below the reference median, and underweight as a weight-for-age Z score of less than negative 2 SD below the reference median [30].

### Exposure variable

Our main exposure variable was women’s empowerment. We followed a method analogous to the SWPER index that was used by Ewerling [11] to compute a standard women’s empowerment scores. The SWPER index was computed based on fifteen items extracted from DHS surveys of 34 African countries following methodical approaches and validated with the existing women’s empowerment measures. The SWPER index better approximated the three (attitude to violence, social independence, and decision-making) dimensions of women’s empowerment [11].

We applied principal component analysis (PCA) on 15 variables that were used to measure women’s empowerment from different perspectives. Before PCA, we re-categorised responses to these items so that higher value was given to categories indicating greater empowerment (Additional file 1: Table S1). Of the 15 components, we retained three principal components that together explained 50% of variations, which were attitude towards violence against women, social independence, and decision-making (named based on what items they were loaded on). These components and the items it contains are presented as a Additional file 1: Table S2.

Most of the identified items were asked only to married or living with a partner (currently married) so that our analyses were restricted to this group. The following three equations used to calculate individual standardized scores both for the composite index and for the three principal components by using the same formula with SWPER computation [11].

1 $${S_{ij}} = \frac{{[[{{\uplambda }_{1j}}({x_{1i}} - {{\bar x}_1})] + [{{\uplambda }_{2j}}({x_{2i}} - {{\bar x}_2})] + \cdots + [{{\uplambda }_{15j}}({x_{15i}} - {{\bar x}_{15}})]]}}{{\sigma_j}}$$

where S_ij_ is the individual standardized score for individual *i* and component *j*; x_1j_,…, x_15j_ are the individual values for variables x_1_–x_15_ included in the PCA analyses; σ_*j*_ is the standard deviation of the predicted scores of each component *j*. The weight given to each of the 15 variables in each component *j* is estimated using Eq. 2 bellow and presented (Additional file 1: Table S3).

2 $${\lambda_{vj}} = \frac{{{\varphi_{vj}}}}{{\sigma_v}}$$

where φ_*vj*_ is the PCA loading for each of the variable *v* in each domain *j* and σ_*v*_ is the standard deviation of each variable *v* in the combined dataset.

Algebraically equation above can be simplified as follow:

3 $${S_{ij}} = \frac{{\left[ { - \left( {\mathop \sum \nolimits_{v = 1}^{15} {\lambda_{vj}}{{\bar x}_v}} \right) + \left( {\mathop \sum \nolimits_{v = 1}^{15} ({\lambda_{vj}}{x_{vi}})} \right)} \right]}}{{\sigma_j}}$$

Based on the above formulas, we can estimate the scores both for the composite index and for the three dimensions of women’s empowerment.

component 1 $$Attitude\;to\;violence\;score = \frac{{\left[ {\left( { - 0.915} \right) + \left( {\mathop \sum \nolimits_{v = 1}^{15} ({\lambda_{v1}}{x_{vi}})} \right)} \right]}}{1.953}$$

component 2 $$Social\;independence\;score = \frac{{\left[ {\left( { - 5.311} \right) + \left( {\mathop \sum \nolimits_{v = 1}^{15} ({\lambda_{v2}}{x_{vi}})} \right)} \right]}}{1.44}$$

component 3 $$Decision\;making\;score = \frac{{\left[ {(0.796) + \left( {\mathop \sum \nolimits_{v = 1}^{15} ({\lambda_{v3}}{x_{vi}})} \right)} \right]}}{1.283}$$

where *x*_vi_ is the value of variables *v* for each individual *i* and λ_*v*0_ − λ_v3_ are the variable weights that are found in the Additional file 1: Table S3. The standardization was made in such a way that it will have the properties of a standard normal distribution with mean = 0 and standard deviation = 1.

Besides, based on available evidence, other child-related factors: acute respiratory infection and diarrhoea status, sex, age and the inter-birth interval [31, 32]; maternal-related factors: height, body mass index and smoking status [33]; and household and environmental-related factors: the source of drinking water, status of sanitation facility, types of cooking fuel, household wealth quintiles and place of residence [34] were adjusted in the analysis. Details on these covariates submitted as an additional file (Additional file 1: Table S4).

### Statistical analysis

The weighted descriptive statistics were generated both for the dependent and independent variables. Bivariate and multivariable logistic regressions were used to assess the association between women’s empowerment and stunting, wasting and underweight in children. As a result of a multistage cluster sampling approach used by the EDHS program, the data obtained from this program form hierarchy in which women nested within a household and households nested within a cluster. This sampling approach allows possibilities in which households from the same cluster may exhibit characteristics that are correlated with one another because of similar contextual conditions. For example, availability and accessibility of food could not be similar across clusters so that those children from the same cluster could have identical nutritional status than those from another cluster. Therefore, in addition to the conventional logistic regression models, we fitted a multilevel logistic regression with two levels that accounted the cluster level random effects (allowing dependence of child nutritional status within a cluster) by using melogit STATA command. The aforementioned covariates were adjusted for both the conventional and the multilevel logistic models. In the multilevel model, we first fitted model 1 to assess the level of intra-cluster correlation, and model 2 (crude model) to assess the association between women’s empowerment and fuel use (i.e. adding each domain into an empty model). Then, model 3 was fitted to examine the independent effect of each empowerment domain controlling for other covariates.

Estimates were presented as adjusted odds ratio (AOR) with 95% confidence intervals (CI) and expressed per one standard deviation increase in each empowerment domain (when women’s empowerment in a specific domain moves away from the average/mean empowerment [which was zero in our case] of the normally distributed population by one standard deviation). A significant association was declared at a p-value of less than 0.05.

## Results

### Weighted descriptive statistics

The total study population comprised of 9998 children, aged 0–5 years. Of these, 5207 (51.1%) children were male. Of the 8877 children, for whom height and age were recorded, 3405 (38.4%) were stunted. Among 9,037 children for whom weight and age were recorded, 2,152 (23.8%) were underweight. Of the 8895 children, for whom weight and height were recorded, 892 (10.0%) were wasted. A total of 1096 (11.6%) and 632 (6.7%) mothers/caregivers reported that their children had diarrhoea and acute respiratory infection (ARI), respectively, in the last two weeks preceding the survey. Short birth-interval (less than 34 months between successive births) was reported in 3801 (38.0%) of the births. Less than one-tenth, 937 (9.4%), of households had improved sanitation facility, 5534 (55.3%) households had improved source of drinking water, and only 303 (3.0%) households used clean fuel for cooking. Only 1032 (10.3%) of households were urban dwellers (Table 1).

**Table 1:** Weighted descriptive statistics of child nutritional status and other individual and household characteristics, 2016 EDHS

Variables	Categories	N (%)
*Child-related factors*		
Stunting (Height-for-age) (n = 8877)	Not stunted	5473 (61.6)
	Stunted	3404 (38.4)
Wasting (Weight-for-height) (n = 8895)	Not wasted	8003 (90.0)
	Wasted	892 (10.0)
Underweight (Weight-for-age) (n = 9037)	Normal	6885 (76.2)
	Underweight	2152 (23.8)
Sex of the child (n = 9998)	Male	5207 (51.1)
	Female	4791 (48.9)
Current age of the child in years (n = 9450)	< 1	2076 (22.0)
	1	1800 (19.0)
	2	1766 (18.7)
	3	1827 (19.3)
	4	1981 (21.0)
Inter-birth interval (n = 9998)	Not short	6197 (62.0)
	Short (< 34 months)	3801 (38.0)
*Health status of children*		
ARI in the last two weeks (n = 9450)	No	8818 (93.3)
	Yes	632 (6.7)
Diarrhea in the last two weeks (n = 9450)	No	8354 (88.4)
	Yes	1096 (11.6)
*Maternal related factors*		
Stature (height) (n = 9997)	Not short (≥ 145 cm)	9772 (97.8)
	Short (< 145 cm)	226 (2.2)
BMI (weight/height) (n = 9998)	Thin	522 (5.2)
	Normal	8872 (88.8)
	Overweight/obese	604 (6.0)
Cigarette smoking status (n = 9998)	No	9921 (99.2)
	Yes	77 (0.8)
*Household factors*		
Source of drinking water (n = 9998)	Improved	5534 (55.3)
	Not improved	4464 (44.7)
Sanitary facility (n = 9998)	Improved	937 (9.4)
	Not improved	9061 (90.6)
Cooking fuel (n = 9998)	Clean	303 (3.0)
	Polluting	9695 (97.0)
Household wealth index (n = 9998)	Poorest	2353 (23.5)
	Poorer	2336 (23.4)
	Middle	2104 (21.4)
	Richer	1828 (18.0)
	Richest	1377 (13.7)
Place of residence (n = 9998)	Rural	8966 (89.7)
	Urban	1032 (10.3)

*ARI* acute respiratory infection, *BMI* body mass index, *cm* centimetre

Table 2 presents the descriptive statistics of the domains of women’s empowerment (Table 2). The descriptive statistics of each of the variable used to compute women’s empowerment is attached as a Additional file 1: Table S3.

**Table 2:** Descriptive statistics of the domains of women’s empowerment, 2016 EDHS

Statistics	Domains of women’s empowerment		
	Attitude to violence	Social independence	Decision-making
Mean	0.00	0.00	0.00
Standard deviation	1.00	1.00	1.00
Minimum	− 1.52	− 2.45	− 3.75
Maximum	1.54	6.41	0.43
Frequency	9914	9914	9914

Values of women’s empowerment domains were standardized, with mean of 0 and SD of 1

### Association of women’s empowerment and child growth faltering

In the unadjusted analysis, all the domains of women’s empowerment were significantly negatively associated with stunting and underweight. Also, the attitude towards domestic violence against women and the social independence domains were significantly negatively associated with wasting (Table 3).

**Table 3:** Fixed and random effect of the model to assess the relationship between women’s empowerment and child growth faltering in Ethiopia, 2016 EDHS

Domains of women’s empowerment	Growth faltering indicators (child nutritional status)					
	Stunting (n = 8076)		Wasting (n = 8142)		Underweight (n = 8241)	
	Crude odds ratio	*P* value	Crude odds ratio	*P* value	Crude odds ratio	*P* value
Attitude to violence	0.85 (0.71, 0.88)	< 0.001	0.91 (0.86, 0.97)	0.004	0.84 (0.80, 0.88)	< 0.001
Social independence	0.81 (0.78, 0.86)	< 0.001	0.86 (0.81, 0.93)	< 0.001	0.77 (0.73, 0.81)	< 0.001
Decision-making	0.86 (0.81, 0.91)	< 0.001	0.95 (0.87, 1.03)	0.213	0.82 (0.77, 0.88)	< 0.001

	Adjusted odds ratio	*P* value	Adjusted odds ratio	*P* value	Adjusted odds ratio	*P* value
	^1^Multivariable logistic regression without random effect^R^					
Attitude to violence	0.92 (0.88, 0.96)	< 0.001	0.96 (0.90, 1.03)	0.333	0.93 (0.89, 0.98)	0.008
Social independence	0.95 (0.89, 0.99)	0.049	0.95 (0.88, 1.03)	0.494	0.91 (0.86, 0.97)	0.002
Decision-making	0.93 (0.87, 0.99)	0.023	1.03 (0.95, 1.13)	0.246	0.92 (0.86, 0.99)	0.020
	^2^Multivariable logistic regression with cluster-level random effect^R^					
Attitude to violence	0.91 (0.87, 0.96)	< 0.001	0.97 (0.90, 1.04)	0.371	0.93 (0.89, 0.99)	0.015
Social independence	0.96 (0.90, 1.01)	0.138	0.96 (0.88, 1.04)	0.299	0.92 (0.86, 0.99)	0.017
Decision-making	0.94 (0.88, 1.01)	0.088	1.01 (0.92, 1.11)	0.782	0.92 (0.85, 0.98)	0.017

^R^Adjusted for child-related factors (sex, age, acute respiratory infection, diarrhoea, and birth interval), maternal factors (body max index, stature (height), and smoking status), household and environmental-related factors (source of drinking water, sanitation facility, type of cooking fuel, wealth index, and location of the cluster), and to one another

^1^Model without random effect, but used CI that accounted for the highest level of clustering (e.g. survey cluster)

^2^Model with cluster-level (primary sampling point) random effect

In the adjusted analysis (model without cluster-level random-effect), the odds of child stunting were 8% lower for each standard deviation increase in women’s attitude towards domestic violence (AOR = 0.92; 95% CI 0.88–0.96; *p* < 0.001). The odds of stunting were 5% and 7% lower for each standard deviation increase in the social independence and the decision-making domains of women’s empowerment (AOR = 0.95; 95% CI 0.89–0.99; *p* = 0.049), and (AOR = 0.93; 95% CI 0.87–0.99; *p* = 0.023), respectively. A one standard deviation increase in the domain of attitude towards domestic violence against women resulted in a 7% reduction in the odds of having an underweight child (AOR = 0.93; 95% CI 0.89–0.98; *p* = 0.008). For each standard deviation increase in social independence and decision-making domains of women’s empowerment, the odds of having an underweight child decreased by 9% (AOR = 0.91; 95% CI 0.86–0.97; *p* = 0.002) and 8% (AOR = 0.92; 95% CI 0.86–0.99; *p* = 0.020), respectively (Table 3).

In the model with a cluster-level random effect term, the only women’s empowerment domain that significantly associated with child stunting was the attitude towards domestic violence against women (AOR = 0.91; 95% CI 0.87–0.96; *p* < 0.001). In the same model, all domains were significantly negatively associated with having an underweight child. For example, as one standard deviation increased in the attitude towards domestic violence against women, the odds of having underweight child decreased by 7% (AOR = 0.93; 95% CI 0.89–0.99; *p* = 0.015). For each standard deviation increase in the domains of social independence and decision-making, the odds for having underweight child were associated with a 8% decrease for each (AOR = 0.92; 95% CI 0.86–0.99; *p* = 0.017) and (AOR = 0.92; 95% CI 0.85–0.98; *p* = 0.017) (Table 3).

The overall outputs from the two adjusted models (a model without cluster-level random effect and a model with a cluster-level random effect term) is attached as a Additional file 1. In those models, the independent effects of other child-related factors, maternal factors, and household and environmental-related factors are displayed (Additional file 1: Table S3).

## Discussions

Child undernutrition is not only caused by the lack of food but can also be affected by other factors, including women’s empowerment [35]. As underscored by the findings of this study, in order to reach optimal nutritional status, women need to feel empowered as they may have a high influence on food selection, procurement, preparation and feeding for children to improve their nutritional status [36]. Previous studies have noted that women’s empowerment reduces the time allocated to childcare and child feeding which, in turn, can lead to poor nutritional outcomes for children [37]. On the other hand, women’s decision-making power can have a positive impact on reducing childhood stunting in that those who have household decisional role can allocate the available resource and select nutritious food for their children [7, 21, 38].

In this study, attitude towards domestic violence against women (individually), social independence (socially), and decision-making abilities (in the household) were the three dimensions of women’s empowerment that were significantly associated with child undernutrition (stunting and underweight). More specifically, in both models (without and with cluster-level random effects), women’s disapproval attitude towards violence against women was associated with a reduction in child stunting. This adds to the remarkable body of evidence on the positive impact of women’s disapproval attitude towards violence on the reduction of child stunting in developing counties where there is very limited evidence (e.g. Ethiopia). This finding could be explained by considering the traditional gender roles that are largely present within Ethiopia. It could be argued that because women are the primary caretakers of children in the household, they could likely have the bargaining power to influence daily household purchases, including variety and adequate food, and appropriate child feeding and childcare practice [23, 36].

In the model without cluster-level random effects, an increase in women’s social independence was associated with a decrease in childhood stunting. This result confirms that women’s social status impacts child nutritional status. This finding has paramount public health importance in countries like Ethiopia because gender norms are existed and practiced in women’s daily life and there are social and cultural norms that restrict women's ability to make choices [39, 40]. In agreement with our finding, a literature review on associations between women’s autonomy and child nutrition by Carlson et al. 2015, indicated a significant association between social independence and childhood stunting [41]. For example, women’s liberty of movement and social support were inversely associated with childhood stunting in Andhra Pradesh, India [42], rural Nicaragua [43], and Burkina Faso [17]. Similarly, the literature review also reported a significant association between women’s freedom of movement and reduction of child stunting [41]. The possible explanation for this could be that when they are socially independent, women have more chance to participate in social events, where they may share child feeding information, and have the ability to justify expenditure on child food. Overall, this can have a profound effect on child nutritional status.

Additionally, we found that one standard deviation increase in women’s decision-making domain was associated with a 7% reduction in the odds of stunting. However, previous studies have reported varied results [23, 37, 44]. For example, in line with our finding, Imai et al. [45], Alkire et al. [46] and Salawu et al. [37] reported a significant association between women’s decision-making power and the reduction of childhood stunting. On the contrary, women’s decision-making power was not significantly associated with childhood stunting in Mozambique [23] in Lao People’s Democratic Republic [47]. Also, being decision-makers in the household had no association with child malnutrition in Pakistan [44]. Regardless of these variations, it is important to note that when women have decisional autonomy on household income allocation, daily household purchase and freedom of movement, they could have opportunity to select and feed their children adequately, and this can lead to potential childhood stunting.

Concerning child’s weight-for-age (underweight) status, we found that having disapproval attitude towards violence against women was negatively associated with childhood underweight. This finding is consistent with previous research reported from Bangladesh [48] and evidence from demographic and health surveys from developing countries [49]. One interpretation for these finding is that the acceptance of violence against women could allow their partners to dominate household resource and suppress women’s decisional role including right to allocate income, purchase items and child feeding practices. As such, women may feel forced to resort to child feeding practices, which are readily available at home, less expensive and also less nutritious.

Moreover, an increase in women’s social independence and participation in decision-making was associated with a decrease in the chance of having an underweight child. In line with our finding, women’s freedom of movement and independence were linked with child underweight in India [38]. If women participated in decision-making at household, the chance of having underweight child decreased [50]. Additionally, even though it was not linked to a specific domain of women’s empowerment, Imai et al. reported the association between women’s empowerment and child underweight [45]. The fact that socially independent women have a chance to meet others and share knowledge, and those who have decisional role could allocate adequate resources, could be one potential explanation of our finding.

In the cluster-level random effect model, the only women’s empowerment domain that significantly and inversely associated with childhood stunting was women’s attitude towards domestic violence. In the same model, all three domains of women’s empowerment were inversely associated with being underweight. Given that the statistical model considered variation (i.e., allowed dependence/similarity of the outcome within the same cluster), this finding points to the idea that children’s nutritional status and women’s empowerment could vary from place to place. The concept is even more important in Ethiopia because there are substantial places of residence where information on child feeding practice and importance is hard to reach. Moreover, gender imbalances exist in different aspects including in the division of labour, access to resources, distribution of income, decision-making and cultural norms that favour toward males in the country.

Finally, results from both models (with and without cluster-level random effects) demonstrated that the three domains of women’s empowerment had no association with wasting in children. As mentioned above, increasing women’s empowerment in all these dimensions would improve child nutritional outcomes and initiate measures to accelerate progress in line with the sustainable development goals.

## Limitation of the study

Despite its strengths, this study has some limitations that must be acknowledged. Although our study employed national representative data, the index that we applied did not include all the variables that could measure women’s status (e.g. ownership status of women on properties such as land and house). Furthermore, most of the woman’s status indicators collected by the DHS applied to only married or currently married women and missed single, widowed, divorced, and separated women. Authors also acknowledge the limitations associated with self-reported data and the limitations that the multivariable model may have missed some variables that could potentially have been relevant.

## Conclusions

This is one of the few studies that shed light on the understanding of child nutrition and women’s empowerment in a rapidly developing country like Ethiopia. Ensuring women’s empowerment both in the household and in the community could have a potential to decrease stunting and underweight in Ethiopia. Policymakers and health professionals need to consider women’s empowerment in this unique context to improve nutritional outcomes for children and alleviate growth faltering.

## Supplementary Information

**Additional file 1: Table S1.** Coded/scaled items used in the development of woman’s empowerment index. **Table S2.** Domains of women’s empowerment with items in the component. **Table S3.** List of variables used in the PCA, component loadings and weight of each variable and all items required to compute a standard score of woman’s empowerment in each component/domain. **Table S4.** Details of the variables included in the regression models.

## Data Availability

The datasets used and/or analyzed during this study can be accessed upon reasonable request from the DHS program website (available at http://dhsprogram.com).

## Abbreviations

ARI: Acute Respiratory Infection
AOR: Adjusted Odds Ratio
CI: Confidence Interval
DHS: Demographic Health Survey
PCA: Principal Component Analyses
SWPER: Survey-based Women’s emPowERment index
SD: Standard Deviation
