# Supplementary material for: Women’s empowerment and child growth faltering in Ethiopia: evidence from the Demographic and Health Survey
Source: BMC Womens Health. 2021 Jan 30;21:42. doi: 10.1186/s12905-021-01183-x (PMC7847175; doi:10.1186/s12905-021-01183-x)
Supplement: Supplementary file 1 — Additional file 1: Table S1. Coded/scaled items used in the development of woman’s empowerment index. Table S2. Domains of women’s empowerment with items in the component. Table S3. List of variables used in the PCA, component loadings and weight of each variable and all items required to compute a standard score of woman’s empowerment in each component/domain. Table S4. Details of the variables included in the regression models. [file 12905_2021_1183_MOESM1_ESM.docx]

**Additional file 1: Table S1.** Coded/scaled items used in the development of woman’s empowerment index

| **Variable name** | **Coding/scale assigned** |
| --- | --- |
| Woman's education in completed years of schooling | Years |
| Frequency of reading newspaper or magazine | Not at all=0, <once a week=1,  ≥once a week=2 |
| Education difference: woman's minus husband's completed years of schooling | Years |
| Respondent worked in past 12 months | No=0, in past year=1, have a job, but on leave past 7 days=2; currently working=2 |
| Age difference: woman's age minus husband's age | Years |
| Age at first cohabitation | Years |
| Age of woman at first birth | Years |
| Decision on respondent's health care | Husband or other alone= –1,  joint=0, respondent alone=1 |
| Decision on large household purchases | Husband or other alone= –1,  joint=0, respondent alone=1 |
| Decision on visits to family or relatives | Husband or other alone= –1,  joint=0, respondent alone=1 |
| Beating justified if wife goes out without telling husband | Justified= –1; don't know=0;  not justified =1 |
| Beating justified if wife neglects the children | Justified= –1, don't know=0,  not justified=1 |
| Beating justified if wife argues with husband | Justified= –1, don't know=0,  not justified=1 |
| Beating justified if wife refuses to have sex with husband | Justified= –1, don't know=0,  not justified=1 |
| Beating justified if wife burns the food | Justified= –1, don't know=0,  not justified=1 |

**Additional file 1:Table S2.** Domains of women’s empowerment with items in the component.

| **Empowerment**  **Domain (as used in SWPER)** | **Variable** | **Code or unit** |
| --- | --- | --- |
| Attitude to  violence | Beating NOT justified if: | Yes = -1;  Don’t know=0;  No=1 |
|  | 1. wife goes out without telling husband |  |
|  | 2. wife neglects the children |  |
|  | 3. wife argues with husband |  |
|  | 4. wife refuses to have sex with husband |  |
|  | 5. wife burns the food |  |
| Social independence | 6. Frequency of reading newspaper or magazine | Not at all=0;  <once a week=1;  ≥once a week=2 |
|  | 7. Woman education in completed years of schooling | Years |
|  | 8. Education difference: woman minus husband completed years of schooling | Years |
|  | 9. Age difference: woman age minus husband age | Years |
|  | 10. Age at first cohabitation | Years |
|  | 11. Age of woman at first birth | Years |
| Decision-making | Who usually decides on:  12. Respondent's health care  13. Large household purchases  14. Visits to family or relatives | Husband/other alone= -1; joint=0;  respondent alone=1 |
|  | 15. Respondent worked in last 12 months | No = 0;  In the past year = 1;  Have a job, but on leave last 7days = 2;  Currently working = 2 |

Note: Category “Don’t know” was set to missing

**Additional file 1:** **Table S3.** List of variables used in the PCA, component loadings and weight of each variable and all items required to compute standard score of woman’s empowerment in each component/domain

| **Variable** | **Comp1  loading** | **SD** | **weight** | **mean** | **Weight* mean** | **Comp2  loading** | **SD** | **weight** | **mean** | **Weight* mean** | **Comp3  loading** | **SD** | **weight** | **mean** | **Weight* mean** |
| --- | --- | --- | --- | --- | --- | --- | --- | --- | --- | --- | --- | --- | --- | --- | --- |
| Woman education | 0.0867 | 3.89 | 0.022 | 2.25 | 0.050 | 0.3974 | 3.89 | 0.102 | 2.25 | 0.230 | 0.0994 | 3.89 | 0.026 | 2.25 | 0.057 |
| Reading frequency | 0.0491 | 0.34 | 0.144 | 0.09 | 0.013 | 0.3321 | 0.34 | 0.977 | 0.09 | 0.088 | 0.065 | 0.34 | 0.191 | 0.09 | 0.017 |
| Education difference | -0.0324 | 3.67 | -0.009 | -1.46 | 0.013 | 0.1346 | 3.67 | 0.037 | -1.46 | -0.054 | 0.0442 | 3.67 | 0.012 | -1.46 | -0.018 |
| Respondent work | -0.0103 | 0.87 | -0.012 | 0.66 | -0.008 | 0.1224 | 0.87 | 0.141 | 0.66 | 0.093 | 0.1172 | 0.87 | 0.135 | 0.66 | 0.089 |
| Age deference | 0.0175 | 7.31 | 0.002 | -8.03 | -0.019 | 0.1123 | 7.31 | 0.015 | -8.03 | -0.123 | -0.0029 | 7.31 | 0.000 | -8.03 | 0.003 |
| Age at first birth | -0.0309 | 3.62 | -0.009 | 19.03 | -0.162 | 0.5822 | 3.62 | 0.161 | 19.03 | 3.061 | -0.0444 | 3.62 | -0.012 | 19.03 | -0.233 |
| Age at 1^st^ cohabitation | -0.021 | 3.62 | -0.006 | 19.03 | -0.110 | 0.5877 | 3.62 | 0.162 | 19.03 | 3.089 | -0.0443 | 3.62 | -0.012 | 19.03 | -0.233 |
| Decision on health | -0.0085 | 3.73 | -0.002 | 17.14 | -0.039 | -0.0191 | 3.73 | -0.005 | 17.14 | -0.088 | 0.5912 | 3.73 | 0.158 | 17.14 | 2.717 |
| Decision on purchase | -0.0138 | 0.61 | -0.023 | -0.04 | 0.001 | -0.0105 | 0.61 | -0.017 | -0.04 | 0.001 | 0.5632 | 0.61 | 0.923 | -0.04 | -0.037 |
| Decision on visit | 0.0157 | 0.59 | 0.027 | -0.13 | -0.003 | -0.022 | 0.59 | -0.037 | -0.13 | 0.005 | 0.5463 | 0.59 | 0.926 | -0.13 | -0.120 |
| Beat if goes out without telling | 0.443 | 0.63 | 0.703 | 0.03 | 0.021 | -0.0099 | 0.63 | -0.016 | 0.03 | 0.000 | -0.0138 | 0.63 | -0.022 | 0.03 | -0.001 |
| Beat if neglect the children | 0.4567 | 1 | 0.457 | 0.04 | 0.018 | -0.0246 | 1 | -0.025 | 0.04 | -0.001 | -0.0002 | 1 | 0.000 | 0.04 | 0.000 |
| Beat if argues with husband | 0.463 | 0.99 | 0.468 | 0.11 | 0.051 | -0.0021 | 0.99 | -0.002 | 0.11 | 0.000 | -0.0218 | 0.99 | -0.022 | 0.11 | -0.002 |
| Beat if refuses to have sex | 0.4219 | 1 | 0.422 | 0.04 | 0.017 | 0.006 | 1 | 0.006 | 0.04 | 0.000 | 0.0006 | 1 | 0.001 | 0.04 | 0.000 |
| Beat if burn food | 0.4354 | 0.99 | 0.440 | 0.12 | 0.053 | -0.0061 | 0.99 | -0.006 | 0.12 | -0.001 | 0.0204 | 0.99 | 0.021 | 0.12 | 0.002 |
|  |  |  |  |  | **-0.105** |  |  |  |  | **6.299** |  |  |  |  | **2.242** |

Comp1- component one, Comp2-component two, Comp3- component three, SD- standard deviation

**Additional file 1: Table S4.** Details of the variables included in the regression models

| Details on the variables included in the regression models | | |
| --- | --- | --- |
| Variables | | **Reference category** |
| Attitude to violence |  | Continuous |
| Social independence |  | Continuous |
| Decision-making |  | Continuous |
| ARI in the last two weeks | Yes |  |
|  | No | No |
| Diarrhoea in the last two weeks: | Yes |  |
|  | No | No |
| Child’s sex | Male | Male |
|  | Female |  |
| Child’s age | < 1 year | <1 year |
|  | 1 year old |  |
|  | 2 years old |  |
|  | 3 years old |  |
|  | 4 years old |  |
| Maternal body mass index | Thin | Thin |
|  | Normal |  |
|  | Overweight/obese |  |
| Maternal stature | normal | Normal |
|  | short (<145cm) |  |
| Source of drinking water | Improved | Improved |
|  | Unimproved |  |
| Sanitation facility | Improved | Improved |
|  | Unimproved |  |
| Type of cooking fuel | Polluting | Polluting |
|  | Clean |  |
| Household wealth index: | poorest | Poorest |
|  | Poorer |  |
|  | Middle |  |
|  | Richer |  |
|  | Richest |  |
| Place of residence | Urban | Urban |
|  | Rural |  |
